# An Auto-Tuning Continuous-Time Bandpass Sigma-Delta Modulator with Signal Observation for MEMS Gyroscope Readout Systems

**DOI:** 10.3390/s20071973

**Published:** 2020-04-01

**Authors:** Chunge Ju, Xiang Li, Junjun Zou, Qi Wei, Bin Zhou, Rong Zhang

**Affiliations:** Department of Precision Instruments, Tsinghua University, Beijing 10084, China; jcg17@mails.tsinghua.edu.cn (C.J.); li-x07@mails.tsinghua.edu.cn (X.L.); zjj19@mails.tsinghua.edu.cn (J.Z.); zhoub@mail.tsinghua.edu.cn (B.Z.); rongzh@tsinghua.edu.cn (R.Z.)

**Keywords:** auto-tuning, bandpass sigma-delta (ΣΔ) modulator, MEMS gyroscopes, MOS resistance

## Abstract

This paper presents the design and implementation of an auto-tuning continuous-time bandpass sigma-delta (ΣΔ) modulator for micro-electromechchanical systems (MEMS) gyroscope readout systems. Its notch frequency can well match the input signal frequency by adding a signal observation to the traditional ΣΔ modulator. The filter of the observation adopts the same architecture as that of the traditional ΣΔ modulator, allowing the two filters to have the same response to input signal change, which is converted into a control voltage on metal-oxide semiconductor (MOS) resistance in the filters. The automatic tuning not only works to solve the mismatch problem caused by process error and temperature variation, but can also be applied to the interface circuit of gyroscopes with different resonant frequencies. The circuit is implemented in a 0.18-μm complementary metal-oxide semiconductor (CMOS) process with a core area of 2.4 mm^2^. The improved modulator achieves a dynamic range of 106 dB, a noise floor below 120 dB and a maximum signal-to-noise and distortion ratio (SNDR) of 86.4 dB. The tuning capability of the chip is relatively stable under input signals from 6 to 15 kHz at temperatures ranging from −45 to 60 °C.

## 1. Introduction

Micro-electromechanical systems (MEMS) gyroscopes are widely used to measure the rotation rate and have many advantages, such as low cost, small size, low power consumption, good complementary metal-oxide semiconductor (CMOS) compatibility, and suitability for batch fabrication [1]. Furthermore, the demand for high-performance micro-machined gyroscopes is growing in platform stabilization, industrial measurement, and many other areas [2,3]. A typical capacitive MEMS gyroscope interface is composed of a capacitance-to-voltage converter (C/V) and an analog-to-digital converter (ADC) [1]. For high precision MEMS gyroscopes, an ADC should have features of high signal-to-noise and distortion ratio (SNDR), a low noise floor, and stable performances under different temperatures and process errors.

Among the various techniques for implementing the inertial sensor digital output, sigma-delta (ΣΔ) modulators are widely used, since they combine the benefits of feedback and inherent ADC [4], which can increase the stability of the system. Additionally, ΣΔ modulators are normally used as high-resolution ADCs with low power. The sampling rate is well above the Nyquist rate to spread the quantization noise over a larger frequency band, and a high amplification in the loop filter causes a suppression of the quantization noise [5].

Since the output of MEMS gyroscopes is a narrowband amplitude-modulated signal, a bandpass ΣΔ modulator is more appropriate than a low-pass ΣΔ modulator [6,7]. The noise is shaped around the notch frequency in the pass-band of the filter. A continuous-time (CT) circuit technique is often applied for the design of the interface application-specific integrated circuit (ASIC) to achieve both a low noise floor and low power consumption [8]. Compared with discrete-time (DT) techniques, it avoids noise folding problems and lowers the requirements for the first operational amplifier in the ΣΔ modulators. Therefore, the CT bandpass ΣΔ modulator is more suitable for the readout circuits of MEMS gyroscopes. A high-order DT force-feedback bandpass ΣΔ modulator was presented by Dong,Y. et al. [7]. This architecture uses a relatively low sampling frequency and thus reduces the requirements for the circuit compared to a low-pass ΣΔ modulator. On the basis of this work, Dong,Y. et al. then proposed a sixth-order CT force-feedback bandpass ΣΔ modulator for the sense mode of MEMS gyroscopes [9].

**Figure 1:**
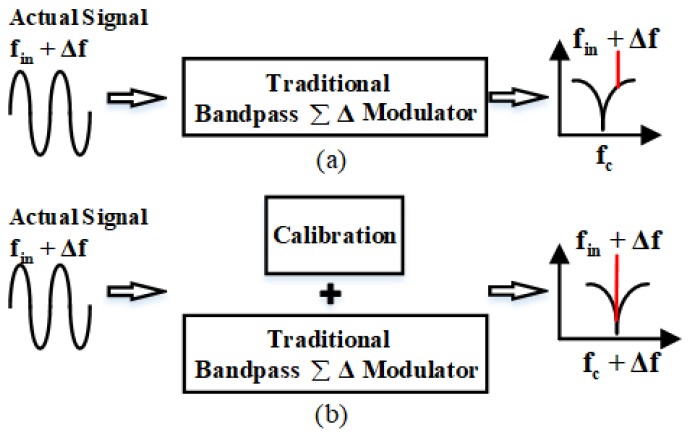
(**a**) The actual signal frequency mismatches the center frequency of the traditional bandpass sigma-delta (ΣΔ) modulator. (**b**) A calibration module added to the bandpass ΣΔ modulator can track the frequency change of the input signal.

However, a CT circuit is sensitive to process, temperature, and voltage variations [10], as filter time constants are a function of resistances or transconductances. Since there is a narrow bandwidth signal in gyroscope readout systems, the relative matching requirements on the electronic filter are stringent [11]. As shown in Figure 1a, the actual signal is affected by the above factors and there is a frequency deviation from the ideal signal. Therefore, for high-precision MEMS gyroscopes, it is important that the notch frequency of the bandpass ΣΔ ADC track the change of MEMS resonant frequency, which is depicted in Figure 1b. Furthermore, due to disturbance of the external environment and long-term drifts, it is only the initial calibration of the frequency matching that does not meet the work requirements. Therefore, it is necessary to tune the ΣΔ ADC center frequency in the whole operation process with an online calibration module that is able to work continuously [11]. 

To avoid the mismatch problems of *RC* times or transconductors, a method of using replica circuits is commonly used. The systems in [12,13] use the same capacitor array in the CT integrator and the calibration circuit. In the calibration circuit, a new voltage signal, depending on the capacitor, is compared with the reference voltage. Then a control capacitor code is generated and sent back to the CT integrator. This method limits the continuity of tuning and introduces parasitic capacitance and resistance. The system presented in [14,15] uses test signals to achieve mode matching. However, the module of signal tone generation is off-chip with extra area. Another tuning system is described in [11] based on noise observation, which adds an area to compare the noise power in two bands located symmetrically above and below the node point. However, the signal spectra cannot be of ideal symmetry and the correction method is composed of multiple modules that are complex to design. The tuning schemes are implemented by changing the transconductance in an operational amplifier, which affects the linearity of the system [13,16]. 

In this paper, a novel automatic tuning system based on metal-oxide semiconductor (MOS) resistance is presented to lower noise floors and to improve the adaptability to temperature drifts and process errors. An on-chip signal observation works overhead with a replica of a loop filter in the ΣΔ modulator to solve the filter tuning problem, which can increase frequency range and tuning accuracy. The measurement results indicate that the tuning range of the bandpass ΣΔ modulator is from 6 to 15 kHz at a temperature ranging from −45 to 60 °C, thus allowing it to be applied to the interface circuit of gyroscopes with different resonant frequencies. The specifications and principles of the background frequency tuning are explained in Section 2; the design of the interface ASIC is shown in Section 3; and Section 4 presents the measurement results of the frequency tuning circuit.

## 2. Basic Principles of Auto-tuning

**Figure 2:**
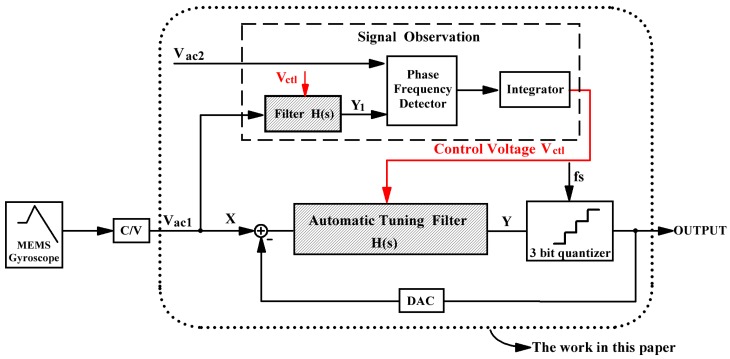
Schematic of the frequency tuning based on ΣΔ modulator for micro-electromechchanical systems (MEMS) gyroscope readout systems. The work of this paper in the rounded rectangle realizes the complete on-chip integration.

The block diagram of the automatic tuning based on the ΣΔ modulator is shown in Figure 2. The system consists of a conventional CT second-order ΣΔ modulator with a 3-bit quantizer and a signal observation. The control voltage $V_{ctl}$ which is generated by the signal observation can tune the time constants of the electronic filters to further change the center frequency of the bandpass ΣΔ modulator.

The following describes the working process of the automatic tuning. The common input signal $V_{ac1}$ flows into the two filters with the same open-loop transfer function H(s). The structure and parameters of the loop filter in the signal observation are the same as those of the automatic tuning filter in the ΣΔ modulator, which are represented by the filled in slashes in Figure 2. In the same chip, the two signals Y and $Y_{1}$ are almost affected to the same extent by an ASIC process error and temperature variations. Another input signal, $V_{ac2}$, with the same frequency as $V_{ac1}$, is a standard signal that is supplied externally. The signals $Y_{1}$ and $V_{ac2}$, with the same frequency, flow into the phase frequency detector (PFD). The PFD can generate different voltage values according to the signal frequency and the phase difference between the two signals. The output voltage of the PFD passes through an integrator to eliminate voltage ripple. Finally, the integrator generates the control voltage signal $V_{ctl}$, which is applied to the tunable voltage-mode MOS resistances in the loop filters. The control voltage $V_{ctl}$ is applied to the two loop filters simultaneously to keep the same H(s). 

According to the principle of automatic tuning, the interface ASIC with a signal observation can track and adjust the notch center frequency for gyroscopes of different resonant frequencies, and can have a good process and environmental adaptability. The following content in this section introduces the module design related to auto-tuning.

### 2.1. CT Bandpass ΣΔ Modulator Archiecture

**Figure 3:**
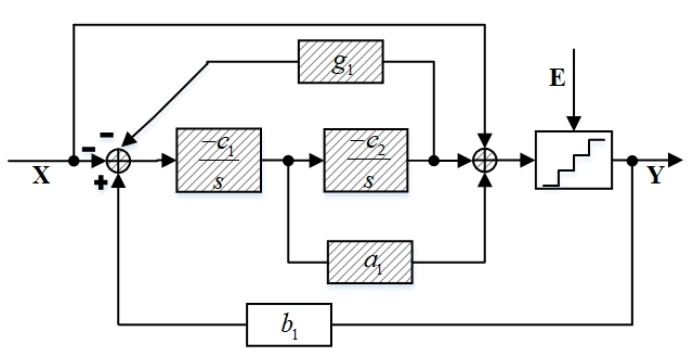
Architecture of the single-loop second-order ΣΔ modulator.

The bandpass ΣΔ modulator employs a single-loop second-order ΣΔ modulator with a 3-bit quantizer using feedforward and feedback paths, as shown in Figure 3. This work uses a second-order ΣΔ architecture because it is stable and easy to design for the observation. Meanwhile, a 3-bit quantizer is used for its advantages of enhancing dynamic range and decreasing quantization noise. The signal transfer function (STF) and the noise transfer function (NTF) of the system in Figure 3 are as follows:

(1) $$STF=\frac{Y}{X}=\frac{s^{2}+a_{1}c_{1}s+c_{1}c_{2}+c_{1}c_{2}g_{1}}{s^{2}+a_{1}b_{1}c_{1}s+b_{1}c_{1}c_{2}+c_{1}c_{2}g_{1}}$$

(2) $$NTF=\frac{Y}{E}=\frac{s^{2}+c_{1}c_{2}g_{1}}{s^{2}+a_{1}b_{1}c_{1}s+b_{1}c_{1}c_{2}+c_{1}c_{2}g_{1}}$$

### 2.2. Loop Filter Architecture in Signal Obsesrvation

**Figure 4:**
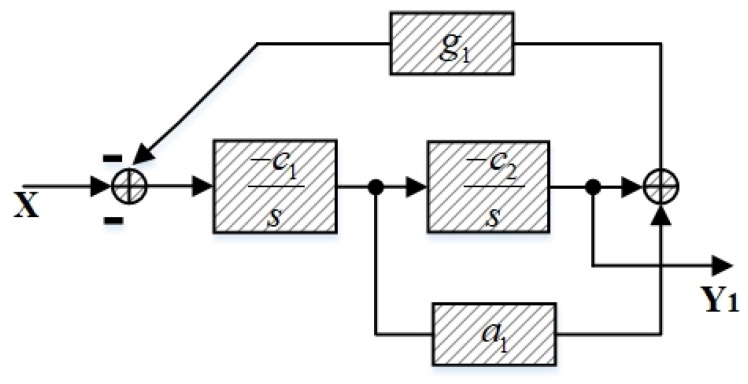
Architecture of the loop filter in the signal observation.

The core of the signal observation is the design of the loop filter. As is described above, the architectures of the two loop filters in the ΣΔ modulator and the signal observation are identical. In Figure 3 and Figure 4, the same modules and parameters are filled with slashes. If the signal observation and the ΣΔ modulator have different loop filter architectures, the operational amplifier and passive devices will be different and will introduce different nonlinear errors. In addition, different layouts will bring in different parasitic capacitors and resistors. These problems are difficult to correct in different forms of structure. Therefore, the signal observation adopts the same loop filter structure as the ΣΔ modulator, which can reduce the mismatch error caused by non-ideal factors and can further improve SNDR.

The transfer function of the observation system is expressed as

(3) $$TF=\frac{Y_{1}}{X}=-\frac{c_{1}c_{2}}{s^{2}+a_{1}c_{1}g_{1}s+c_{1}c_{2}g_{1}}$$

In this circuit design, all of the electronic filters adopt the same parameters; thus, $c_{1}=c_{2}=c$. When the frequency of the input signal changes, the frequency response of NTF in (2) and TF in (3) will change correspondingly, as shown in expressions (4) and (5). 

**Figure 5:**
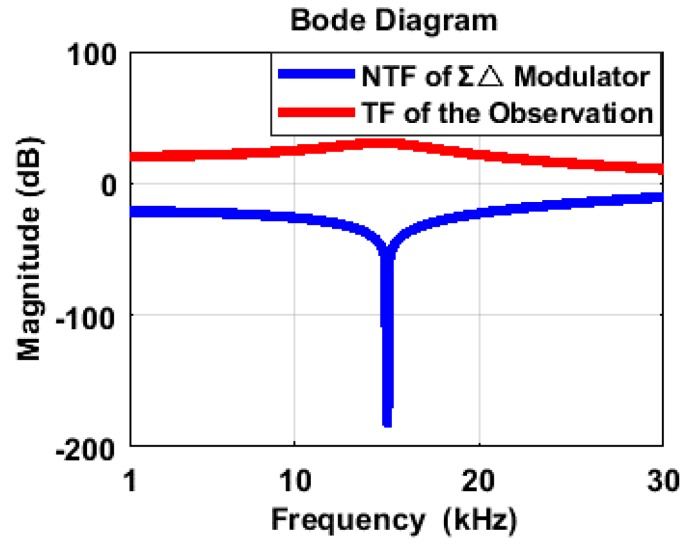
Spectrums of the ΣΔ modulator’s noise transfer function (NTF) and the signal observation’s transfer function (TF).

Figure 5 depicts the frequency response of the transfer functions (4) and (5). Obviously, the corresponding frequency of the peak response in the signal observation is consistent with the notch frequency in the ΣΔ modulator, which is the main principle of the signal observation design.

(4) $$NTF_{1}=\frac{s^{2}+{\left(c+∆c\right)}^{2}g_{1}}{s^{2}+a_{1}b_{1}\left(c+∆c\right)s+b_{1}{\left(c+∆c\right)}^{2}+{\left(c+∆c\right)}^{2}g_{1}}$$

(5) $$TF_{1}=-\frac{{\left(c+∆c\right)}^{2}}{s^{2}+a_{1}g_{1}\left(c+∆c\right)s+{\left(c+∆c\right)}^{2}g_{1}}$$

### 2.3. Control Voltage Generation

**Figure 6:**
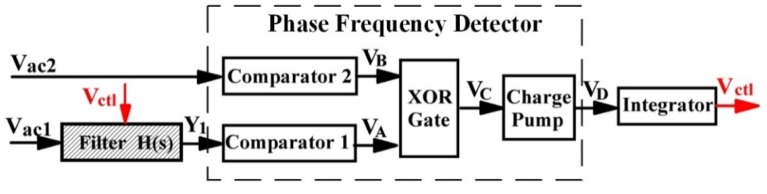
Schematic of the signal observation.

Figure 6 shows the block diagram of the signal observation. $V_{ac1}$ passes through the loop filter before flowing into Comparator 1. $V_{ac2}$ directly flows into Comparator 2 to generate a square wave $V_{B}$. The square wave $V_{A}$ with different phases is derived from Comparator 1, which compares signal $Y_{1}$ with a DC standard voltage. Next, an Exclusive OR (XOR) gate calculates the phase difference between $V_{A}$ and $V_{B}$ to obtain the digital signal $V_{C}$ and the charge pump converts it into an analog signal $V_{D}$. The relation between the value of $V_{D}$ and the phase difference ∆θ is:

(6) $$V_{D}=(1/2\pi K_{cp}s)∆\theta$$

where $K_{cp}$ is the coefficient associated with the charge pump.

Finally, the control voltage $V_{ctl}$ is obtained by the integral operation (7).

(7) $$V_{ctl}=\left(1+1/RCs\right)V_{D}$$

**Figure 7:**
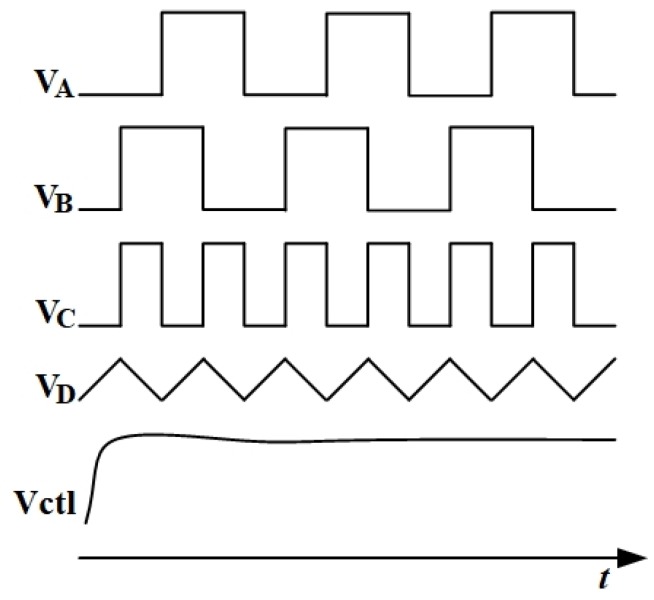
Simplified operation waveforms of the signal observation.

Figure 7 shows the above operation waveforms transformation. The voltage $V_{ctl}$ directly determines the values of the MOS resistances, which means that $V_{ctl}$ determines the time constants of the electronic filters. Furthermore, $V_{ctl}$ can automatically adjust the notch frequency in the ΣΔ modulator.

## 3. Circuit Implementation

In the following section, the transistor-level implementation of the amplifier, the MOS resistance, and the digital-to-analog converter (DAC) in the proposed ΣΔ modulator are explained.

### 3.1. CT Bandpass ΣΔ Modulator Architecture

**Figure 8:**
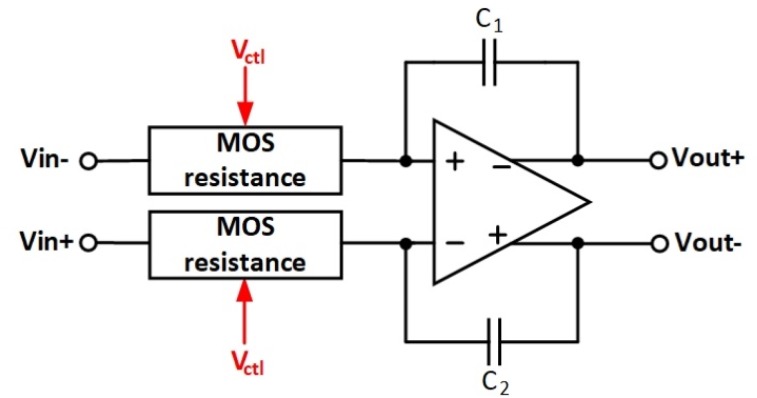
System-level of the low-pass metal-oxide-semiconductor filed-effect transistor-capacitor (MOSFET-C) filter.

**Figure 9:**
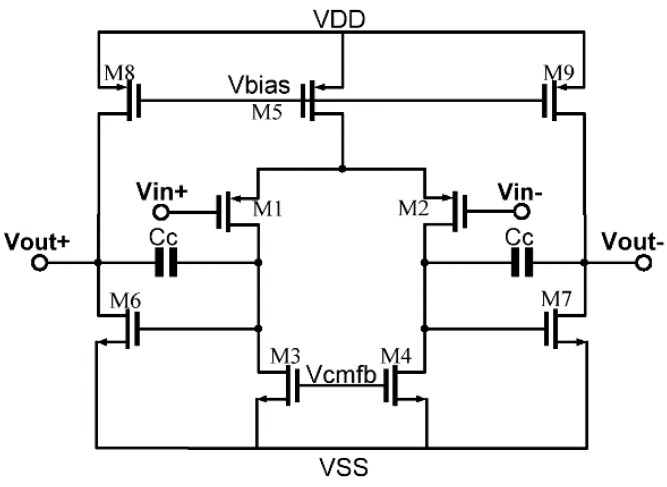
Transistor-level implementation of the amplifier.

To make possible the automatic tuning of the ΣΔ modulator, a metal-oxide-semiconductor filed-effect transistor-capacitor (MOSFET-C) filter is adopted using a tunable voltage-mode MOS resistance, a capacitor, and an operational amplifier, as shown in Figure 8. In order to improve the accuracy of the ADC, the first amplifier requires high open-loop gain and low noise. A transistor-level schematic of the two-stage amplifier is depicted in Figure 9. To decrease 1/f noise, the amplifier adopts PMOS input differential stages, as the 1/f noise of PMOS is lower than NMOS and the gate length of the load transistor should be larger than that of the input transistor [17]. Another design guideline is to maximize the gain of the first stage [18]. The amplifier in Figure 9 can achieve low noise by optimizing the parameters of the transistors. The amplifier performance is given in Table 1. Considering that the resonant frequency of an MEMS gyroscope is generally above 6 kHz, the equivalent input noise of the amplifier was measured at 6 kHz.

### 3.2. MOS Resistance

**Figure 10:**
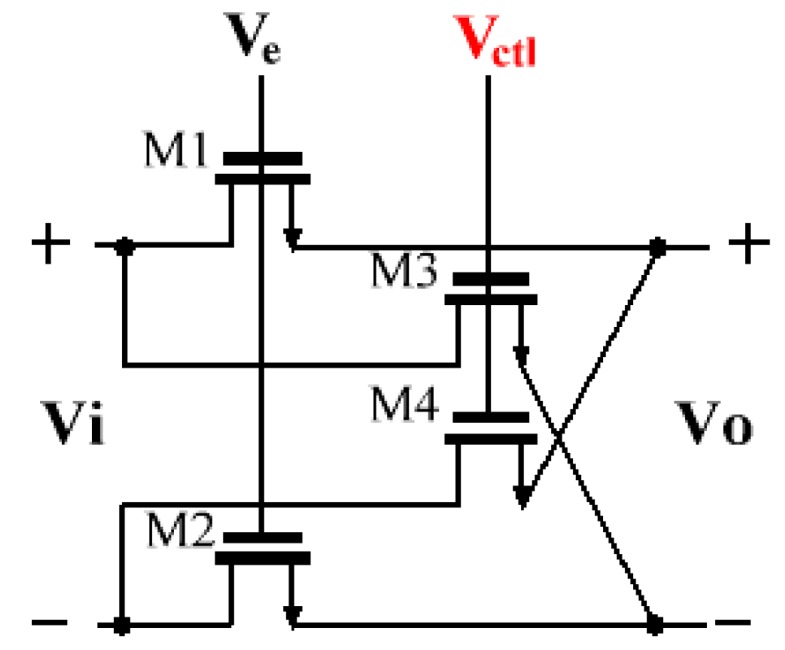
Transistor-level schematic of the differential tunable metal-oxide semiconductor (MOS) resistance.

Figure 10 shows the schematic of the differential tunable voltage-mode MOS resistance in this work, which is implemented by four of the same n-channel MOS transistors (M1, M2, M3, and M4) that work in the triode region. The value of a MOS resistance ($R_{MOS}$) is automatically adjusted through the voltage $V_{e}$ and $V_{ctl}$. Its equivalent resistance is calculated in (8):

(8) $$R_{MOS}=\frac{1}{\mu_{n}C_{ox}W/L\left(V_{ctl}-V_{e}\right)}$$

where $C_{ox}$ is the gate oxide capacitance per unit area, $\mu_{n}$ is the electron mobility in the channel, and *W* and *L* are the channel width and length, respectively [19].

The control voltage $V_{ctl}$ is generated by the signal observation and $V_{e}$ is supplied by an external voltage source. The function of the voltage $V_{e}$ is to externally adjust the value of $R_{MOS}$ to facilitate chip testing. Once determined, it will not change during the whole tuning process. The same control voltage is applied to all of the MOS resistances on the chip, which is to keep the transfer functions of the two loop filters the same. In the case of ideal matching, this structure has high resistance linearity and eliminates the effect of the threshold voltage of the transistor [20,21]. However, there are some obvious linearity problems with using MOS resistances in electronic filters. As the amplitude of the signal increases, the linearity of the MOS resistance will deteriorate and MOS transistors will further fall into the saturation zone with the increase of $V_{DS}$ (the voltage difference between the drain and the source). Despite this, the signal generated by MEMS gyroscopes is extremely small and the magnification factor of C/V can be set to an appropriate value to avoid such a nonlinearity problem.

### 3.3. DAC

**Figure 11:**
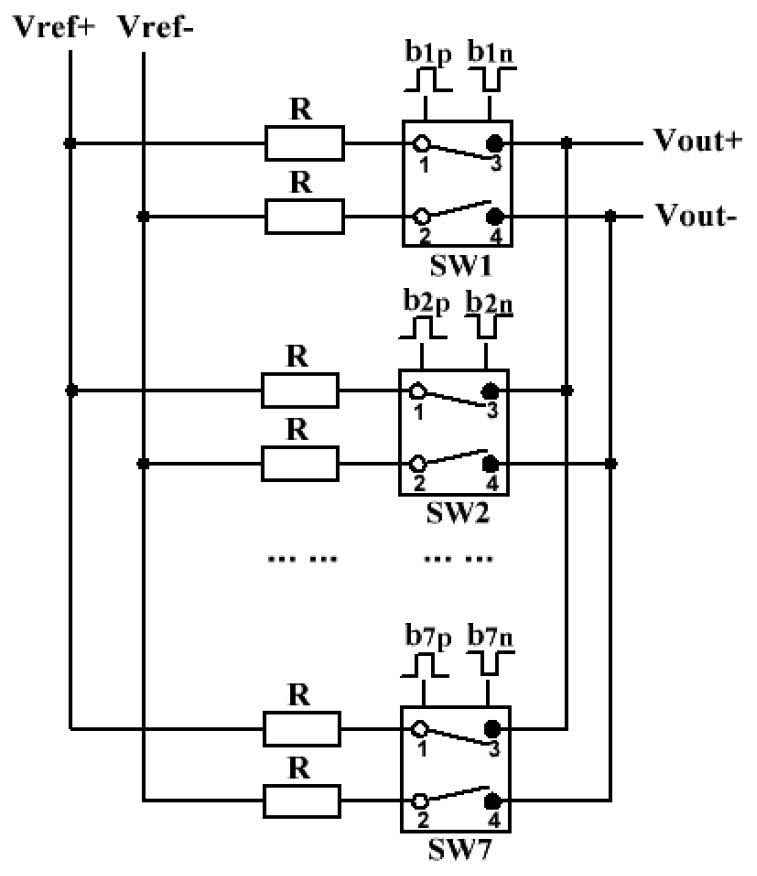
Schematic of the 3-bit resistance-type digital-to-analog converter (DAC).

The DAC shown in Figure 11 is implemented using switching resistance technique. According to the change of the input signal ($b_{1p,n}-b_{7p,n}$), the switch has two working states, namely, are parallel-connected and cross-connected. The output signal of the 3-bit DAC is expressed in (9):

$$V_{out+}=\frac{m\cdot V_{ref+}+\left(7-m\right)\cdot V_{ref-}}{7}$$

(9) $$V_{out-}=\frac{m\cdot V_{ref-}+\left(7-m\right)\cdot V_{ref+}}{7}\;m=1,2\cdots 7$$

where m is the number of the switches when parallel-connected, and $V_{ref+}$ and $V_{ref-}$ are the values of the references of the DAC.

## 4. Measurement Results

**Figure 12:**
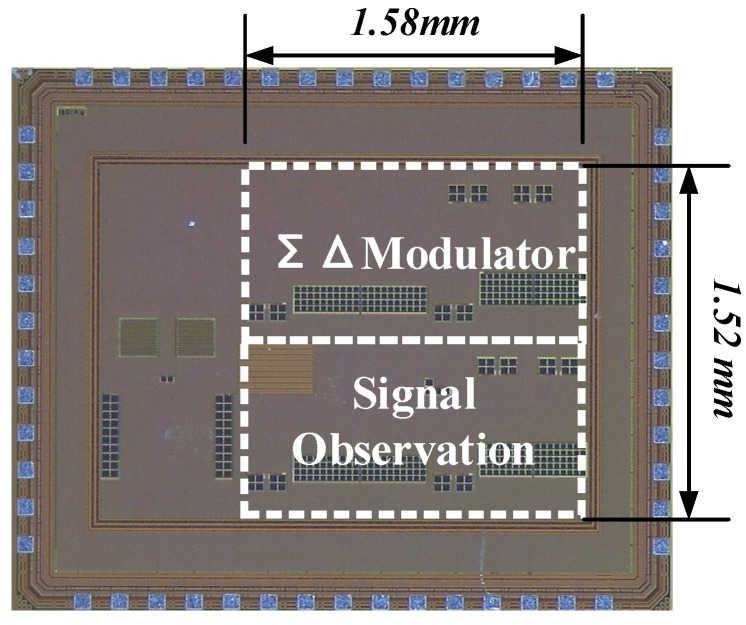
Microphotograph of the ΣΔ modulator with signal observation.

A chip micrograph of the ASIC is shown in Figure 12, which was fabricated in SMIC 0.18-μm Mixed Signal 1P6M COMS process. A 5V power supply voltage helps the gyroscope achieve high precision. In the layout design, the electrical filters in the signal observation and the ΣΔ modulator are located as close to each other as possible. The ASIC occupies 2.4 mm^2^ of the active area and consumes 7.8 mA. The area required for the signal observation is approximately 0.99 mm^2^. The performance of the modulator was evaluated using a high-resolution sinusoidal source as input signals and a digital square wave source as sampling signal. 

**Figure 13:**
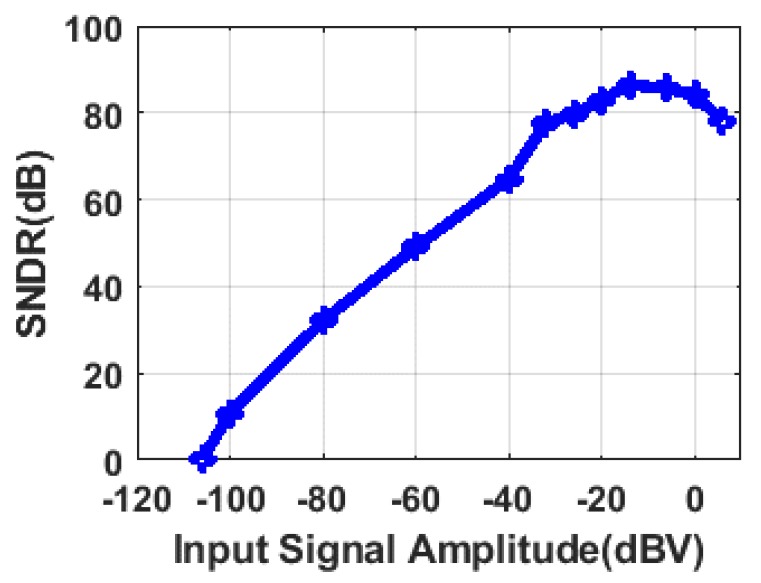
Signal-to-noise and distortion ratio (SNDR) versus 15 kHz input signal amplitude.

The circuit was first tested at 25 °C with a sampling clock $f_{s}$ = 1.5 MHz. Since only narrowband signal is concerned, SNDR is calculated as the output signal power to noise and distortion power in a two-sided bandwidth of 200 Hz. A total of five chips were tested to verify the repeatability of the data. The measured SNDR is presented versus a 15 kHz input signal amplitude in Figure 13. The input-referred dynamic range (DR) is approximately 106 dBV [22], and the maximum SNDR is 86.4 dB when a −14 dBV input signal is applied. MEMS gyroscope interface circuits measure tiny capacitors variations. Since the amplitude of the input signal is small, the performance of SNDR is not better than commercial ADC. However, for gyroscopes, in-band noise (IBN) is more relevant than the overall SNDR. It needs to be noted that the amplitudes of input signals are −26 dBV in the following measured performances.

**Figure 14:**
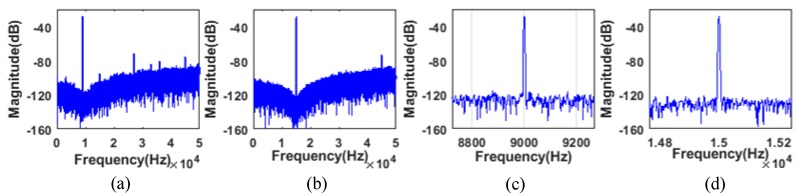
Spectrums of the output signal of the second-order ΣΔ modulator with signal observation. (**a**) A 9 kHz input sinusoidal wave and (**b**) a 15 kHz input sinusoidal wave. (**c**) Partial view of the spectrum in (**a**) near 9 kHz. (**d**) Partial view of the spectrum in (**b**) near 15 kHz.

**Figure 15:**
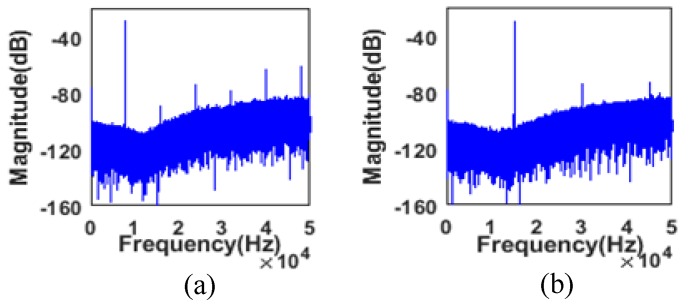
Spectrums of the output signal of the second-order ΣΔ modulator with signal observation off and fixed control voltages when either (**a**) an 8 kHz sinusoidal wave is input or (**b**) a 15 kHz sinusoidal wave is input.

**Figure 16:**
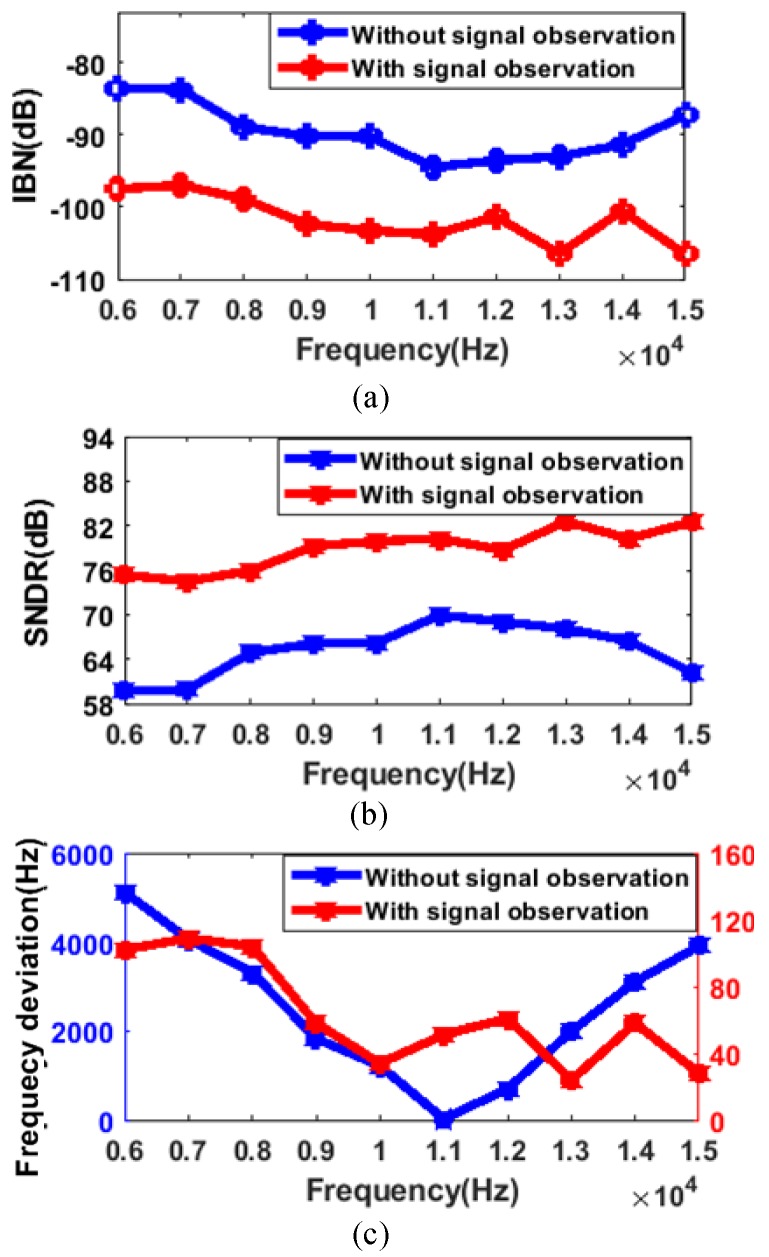
Measured in-band noise (IBN), SNDR, and frequency deviation with disabled and enabled signal observation in (**a**), (**b**,**c**) with input signal frequency ranging from 6 to 15 kHz.

The effect of auto-tuning is illustrated in Figure 14a,b, which shows two output spectrums corresponding to the 9 kHz and 15 kHz input sinewaves. Figure 14c,d shows the partial view of the output spectrum near the resonant frequency, which indicates the noise floor below −120 dB in a 400 Hz bandwidth. In the same situation, the signal observation is turned off and the control voltage is fixed externally to tune the noise shaping notch at 11 kHz. There is a mismatch between the center frequency of the notch and the input signal frequency, as is depicted in Figure 15. This is better illustrated in Figure 16, IBN, SNDR, and frequency deviation ($\left|f_{input\_signal}-f_{notch}\right|$) are shown versus different input signal frequencies with signal observation on and off. IBN calculates the power spectral density within the 200 Hz bandwidth. With signal observation disabled, the performances are significantly better at an input signal of 11 kHz than other input signal frequencies. The maximum IBN deviation is 10.96 dB and the maximum SNDR deviation is 10.28 dB. For frequency deviation, the further the input signal strays from 11 kHz, the larger the deviation. The maximum frequency deviation can be up to 5140 Hz. With signal observation enabled, it can be noted that the IBN is below −96 dB and the SNDR is above 74 dB, with the frequency ranging from 6 to 15 kHz. Due to low-frequency noise, the noise-shaping is slightly degraded when the input signal is below 9 kHz and frequency deviation significantly decreases, with a maximum value of less than 110 Hz. Figure 16a,b and c show that the smaller the frequency deviation, the larger the value of the SNDR and the lower the value of the IBN. A total of five chips were tested to verify the repeatability of the data. This work defines a new parameter SNDR coefficient with frequency (S.CF) to depict the relative change of the SNDR, which is calculated in (10). The S.CF is reduced to 1.13%/kHz with signal observation.

(10) $$S.CF=\frac{SNDR_{max}-SNDR_{min}}{(f_{max}-f_{min})\cdot SNDR_{average}}$$

To verify that temperature variations can be compensated by filter tuning, the ASIC temperature was varied between −45 and 60 °C. Table 2 shows the IBN and the SNDR corresponding to the three different cases of temperature (−45, 25, and 60 °C) with both disabled and enabled signal observation when a 11 kHz input signal is applied. The three temperature cases represent the minimum, normal and maximum temperatures for a chip to work properly. With signal observation disabled, the center frequency of the notch shifted with temperature. The frequency deviation is up to 2230 Hz at −45 °C and 1555 Hz at 60 °C. With signal observation enabled, the notch frequency is basically coincidental with the input signal frequency and the frequency difference is below 0.5% under the above test conditions. The measured IBN is about −100 dB and the SNDR is above 78 dB for each case. From these data, it can be seen that the circuit is stable in the temperature range of −45 to 60 °C. Similar to the definition of S.CF, a parameter SNDR coefficient with temperature (S.CT) is used to describe the relative change of the SNDR with temperature, as calculated in (11). The S.CT has been reduced from 0.091 to 0.023%/°C.

(11) $$S.CT=\frac{SNDR_{max}-SNDR_{min}}{\;\left(T_{max}-T_{min}\right)\cdot SNDR_{average}}$$

Table 3 shows a summary of the performances of the modulator in this work and a comparison with previously reported auto-tuning circuits for CT ΣΔ modulators. For fair comparison, only ΣΔ modulators which were tested or simulated without sensors are shown in Table 3, and [14] is designed for MEMS gyroscope interface circuits. Compared with other works, this work thoroughly tested the auto-tuning ΣΔ modulator at different frequency input signals from 6 to 15 kHz and at different temperatures from −45 to 60 °C. It can reduce the relative variation of the SNDR under different working conditions and can increase the tuning accuracy and tuning range. The value of IBN can be controlled within −96.99 to −103.82 dB, lower than other ΣΔ modulators working in readout systems. Most of the ΣΔ modulators for MEMS gyroscopes do not contain frequency tuning or make frequency adjustment off-chip or off-line. A few of these contain auto-tuning circuits, but do not analyze ΣΔ modulator performance separately [11,12]. 

## 5. Conclusions

This paper demonstrates the significance of incorporating an active tuning module for ΣΔ modulators in MEMS gyroscopes. A second-order bandpass ΣΔ modulator with a tuning circuit was presented, which can track and adjust the notch center frequency for different resonant frequencies. The implementation of the filter tuning uses a signal observation that contains a filter with the same architecture as the loop filter in the traditional ΣΔ modulator, thus both of them have the same response to the input signal change, temperature variation, and process error. In the signal observation, the phase difference of the standard signal and the output signal through the electrical filter is converted into a control voltage applied to the MOS resistances. With the signal observation, the presented ASIC offers a very high flexibility, thus can be used with different gyroscopes. With input signal frequency ranging from 6 to 15 kHz, the signal observation allows the ΣΔ modulator to perform stably at temperatures between −45 and 60 °C. The filter tuning circuit presented is completely implemented on chip and requires an additional area of 0.99 mm^2^. With an appropriate architecture design, the resulted bandpass ΣΔ modulator achieves a DR of 106 dB, a maximum SNDR of 86.4 dB, a tuning accuracy below 0.62% (at 11 kHz input signal), a S.CF of 1.13 %/kHz, and a S.CT of 0.023%/°C. 

## Figures and Tables

**Table 1:** The amplifier performances summary.

Parameters	Values
GBW	188.5 MHz
Av	101.9 dB
${\bar{v}}_{n,input}$ at 6 kHz	$16.53\;\mathrm{nV}/\sqrt{\mathrm{Hz}}$
Current	1.9 mA

**Table 2:** Performances at different temperature points with an 11 kHz sinusoidal input signal.

Temperature (°C)	Without Signal Observation				With Signal Observation			
	IBN (dB)	SNDR (dB)	Frequency Deviation (Hz)	S.CT (%/°C)	IBN (dB)	SNDR (dB)	Frequency Deviation (Hz)	S.CT (%/°C)
−45	−87.62	63.56	2230	0.091	−101.40	78.73	65	0.023
25	−94.50	69.95	61		−103.82	80.26	52	
60	−91.31	66.83	1555		−100.09	78.32	68	

**Table 3:** Performances summary and comparison.

	This Work	[13]	[15]	[16]	[23]
Technology	0.18 μm	0.5 μm	90 nm	1.5 μm	-
Measurement approach	Experiment	Experiment	Simulation	Experiment	Simulation
System architecture	CT second-order Σ△ modulator	CT second-order Σ△ modulator	CT second-order Σ△ modulator	CT fourth-order Σ△ modulator	CT fourth-order Σ△ modulator
Frequency tuning principle	Signal observation	Voltage comparison	Out-of-band tone injection	Charge comparison	Phase detection
Temperature range (°C)	−45~60	Room temperature	Room temperature	Room temperature	−40~27
Frequency range	6~15 kHz	±40%	±40%	-	-
Frequency difference	≤0.62% @ 11 kHz	±10%	1.04%	±1% @10.7 MHz	0.4% @ 10 kHz
SNDR (dB)	86.4 @15 kHz	83 @100 kHz	72.7	68 @10.7 MHz	-
IBN (dB)	−106.57 @15 kHz	-	-	-	−91.3 @10 kHz
Supply (V)	5	3.3	-	±2.5	-
Area (mm^2^)	2.4	0.35 ^1^	-	6	
Power (mW)	39	2 ^1^	-	220	

Note: Only tuning circuit is calculated.
